# The Effect of Edible Mushroom on Health and Their Biochemistry

**DOI:** 10.1155/2022/8744788

**Published:** 2022-03-23

**Authors:** Anmut Assemie, Galana Abaya

**Affiliations:** ^1^Department of Biology, Wachemo University, PO Box 667, Hossana, Ethiopia; ^2^Department of Biotechnology, Wachemo University, PO Box 667, Hossana, Ethiopia

## Abstract

Edible mushrooms are fungi that can be seen with the naked eye and are relatively easy to gather by hand. This review article highlights the health benefit and the biochemistry of several mushroom species. *Agaricus bisporus, Pleurotus species. Lentinus edodes,* and *Volvariella species* are the most acceptable varieties among the cultivated mushroom. Various biochemical methods such as methanol, ethanol, and water extract of different parts of the edible mushroom in the laboratory have been applied to determine and/or quantify the presence and effectiveness of their chemical compounds, food value, and medicinal properties. They contain varying amounts of carbohydrates, proteins, nucleic acids, lipids, minerals, terpenoids, phenolic compounds, steroids, and lectins and vitamins, as well as lowering cholesterol levels in the body. Due to the presence of those vital nutrients, mushrooms are the best food item with high nutritional value. These compounds have a wide range of therapeutic effects and can act as immunomodulatory, anticarcinogenic, antiviral, antioxidant, and anti-inflammatory agents. Routine consumption of edible mushrooms would give adequate protection due to the presence of all the necessary nutrients from them. Therefore, edible mushrooms are herbal antibiotics to many diseases as well as various cancers of humans.

## 1. Introduction

A mushroom is a macro fungus with a distinctive fruiting body and large enough to be seen with the naked eye and to be picked by hand [1]. Mushrooms belong to the family Agaricaceae. The types of mushrooms differ in colors, shapes, surfaces, and activities [2]. They are produced over the ground of soil or on their food sources, such as decaying wood in a moist and cold environment [2].

Of the 1.5 million fungi estimated, 14,000 described species in the world produce fruiting bodies that are large enough to be considered mushrooms [3] and of which at least 2000 species are edible [4]. Of the well-described mushrooms, *Pleurotus, Lactarius, Pisolithus, Tremella, Russula, Agaricus, Lentinus, Auricularia, Hericium, Grifola, Flammulina, and Cordyceps* are well-known edible species [4]. Edible mushroom production occurs by cutting a plant, and some start from the seeds, but development depends on the species. Some mushroom-like elm and oysters are fast-growing as compared to maitake and morels, which take time [5]. Edible mushrooms are collected in the wild or cultivated worldwide [6]. Mushrooms have been widely used as foods [7, 8] and very often as delicious and nutritious foods [9].

Mushrooms contain moisture (85–95%), carbohydrates (35–70%), protein (15–34.7%), fat (10%), minerals (6–10.9%), and nucleic acids (3–8%) [4]. It also contains a large amount of vitamins such as thiamine 1.4–2.2 mg (%), riboflavin 6.7–9.0 mg (%), nacin 60.6–73.3 mg (%), biotin, ascorbic acid 92–144 mg (%), pantothenic acid 21.1–33.3 mg (%), and folic acid 1.2–1.4 mg/100 g in dry weight basis [10]. The minerals that found in mushroom are calcium, iron, manganese, magnesium, zinc, selenium [11]. Since mushrooms have carbohydrates, fiber, protein, essential amino acids, unsaturated fatty acids, vitamins, low calories, and minerals such as potassium, iron, copper, zinc, and manganese, which are high in their fruit bodies, they are recognized as a healthy food with nutritional benefits [12].

Mushroom extracts and their secondary metabolites have acquired antioxidant, antimicrobial, anticancer, anti-inflammatory, antiobesity, and immunomodulatory activities due to their biological effects. Due to this, phytochemists, nutritionists, and consumers are very interested in the phytochemical constituents of mushrooms, which provide beneficial effects to humans in terms of health promotion and reduction of disease-related risks [6]. Polysaccharides, carbohydrate-binding proteins, peptides, proteins, enzymes, polyphenols, triterpenes, and triterpenoids are bioactive compounds derived from mushrooms that are beneficial to human health and have antiviral activity against DNA and RNA viruses [13]. The objective of this review is to understand the nutritional value, biochemistry, and medicinal properties of edible mushrooms.

## 2. Chemicals in Edible Mushrooms

### 2.1. Phenolic Compounds

Phenolic compounds are aromatic hydroxylated compounds with one or more aromatic rings and hydroxyl groups. The anti-inflammatory properties of many mushrooms have been attributed to the presence of some phenolic compounds, which include phenolic acids, hydroxycinnamic acids, lignans, tannins, flavonoids, hydroxybenzoic acids, stilbenes, and oxidized polyphenols [14, 15].

Different scientific research has proven that phenolic compounds have an antifree radical, peroxide decomposers, metal activators, and an oxygen scavenging effect. It also has an effective role in preventing aging and heart disease. Studies have shown that the phenolic compounds in mushrooms have antioxidant properties in addition to being an anticancer agent, as they have been shown to have the ability to inhibit the growth of cancer cells by inhibiting lipopolysaccharide (LPS)-stimulated nitric oxide, in addition to other anticancer mechanisms, including the activation of programmed cell death, and inhibition of mediated reactive oxygen species activity in the NF-B pathway [16].

Edible mushrooms generally do not contain transunsaturated fatty acids, but they do contain ergosterol, as it is used to prevent cardiovascular disease, in addition to being a source for the manufacture of vitamin D [17]. Another type of fatty acid, called tocopherols, has been discovered and is a powerful antioxidant in terms of its ability to free radical scavenging, so it contributes to protecting the heart [18]. Linoleic acid is known for its antioxidant activity, which it achieves by inhibiting the production of nitrous monoxide, tumor necrosis factor, interleukin-6, and interleukin-1. It is therefore used in the treatment of Alzheimer's through its inhibitory activity of acetylcholinesterase and butyrylcholinesterase [19].

The evaluation of total phenolic and flavonoid contents in eight types of edible mushrooms such as *Agaricus bisporus, Boletus edulis, Calocybe gambosa, Cantharellus cibarius, Craterellus cornucopioides, Hygrophorus marzuolus, Lactarius deliciosus,* and *Pleurotus ostreatus* indicate the presence of 1–6 mg of phenolics/g of dried mushroom, and the flavonoid concentrations ranged between 0.9 and 3.0 mg/g of dried matter. The main flavonoids found were myricetin and catechin. In B. edulis and A. bisporus, the highest content of phenolic compounds presented, while *L. deliciosus* showed a high amount of flavonoids and *A. bisporus, P. ostreatus,* and *C. gambosa* presented low levels [20].

### 2.2. Nutritional Values of Edible Mushrooms

Edible mushrooms have been widely utilized as human food for centuries and appreciated for texture, flavor, as well as medicinal and tonic attributes [21].

In general, mushrooms contain 90% water and 10% dry matter [22]. They have a chemical composition, which is attractive from the nutritional point of view [23]. Mushrooms are nutritionally important as they are rich in protein, fibers, and minerals, while poor in fats. The mushroom protein contains all the nine essential amino acids required by humans. Mushrooms are considered as a potential substitute for muscle protein on account of their high digestibility [24]. Besides this, mushrooms are also a rich source of vitamin B1, B2, B12, C, D, and E [25, 26] and a relatively good source of nutrients like phosphorus, iron, and vitamins, including thiamine, riboflavin, ascorbic acid, ergosterol, and niacin [27]. Mushrooms are also an excellent source of vitamin D which is otherwise not available in other food supplements [28].

Mushrooms are low in calories, fat-free, cholesterol-free, gluten-free, and very low in sodium. Minerals such as potassium, iron, copper, zinc, and manganese are high in fruit bodies. They also have ash, glycosides, volatile oils, tocopherols, phenolic compounds, flavonoids, carotenoids, folates, organic acids, etc. [12].

Mushrooms are also important from a nutraceutical point of view, as they contain several compounds like unsaturated fatty acids, phenolic compounds, tocopherols, ascorbic acid, and carotenoids. The nutritional attributes of edible mushrooms and the health-benefiting effects of the bioactive compounds they contain make mushrooms a healthy food [29–31].

Consumers are now deeply interested in food bioactives that provide beneficial effects to humans in terms of health promotion and disease risk reduction. Mushrooms can be considered as functional food which provides health benefits in addition to nutritional value [32]. The concept of “functional foods” was first introduced as a factor in the analysis of foods after nutrients [33].

The nutrient content of edible mushrooms varies depending on the growth and harvest stages and the environment in which they are grown [34]. Edible mushrooms play an important role in human dietary regimen and nowadays, the consumption of these mushrooms has increased. The genus pleuritis consists of almost forty various species that are called oyster mushrooms. The mushrooms are widely consumed because of their flavor, nutritional benefits, and therapeutic characteristics. *P. ostreatus* mushrooms are very important due to their nutraceutical functions and medicinal potential [35]. Edible mushrooms generally have a moisture content of 80% to 90% per 100 grams [36]. To determine the nutritional values and medicinal value of edible mushrooms, researchers use different methods such as crude polysaccharide (LEP) which was extracted by hot water from the fruiting bodies, methanol extract, ethanol extract, water extract (for proteins, lipids, carbohydrates, and vitamins). The nutritional value of edible mushroom shown in Table 1.

### 2.3. Proteins

The crude protein content of edible mushrooms is usually high but varies greatly and is affected by factors such as species and stage of development of the mushroom [38]. Edible mushrooms are a good source of protein, as they contain 200 to 250 grams per kilogram of dry weight. The most common amino acids found in mushrooms are leucine, aspartic acid, valine, glutamine, and glutamic acid. Proteins of Pleurotus species mushroom have higher standard because of efficient dissemination of essential amino acids as well as the nonessential amino acids particularly gamma-aminobutyric acid (GABA), a very important neurotransmitter [6].

### 2.4. Lipids

Edible mushrooms are low caloric food with very little fat (4–6%) and without cholesterol. Total fat content in A. bisporus was reported to be 1.66 to 2.2/100 g on dry weight basis [39, 40]. The most important fatty acids found in edible mushrooms include linoleic acid, oleic acid, and palmitic acid. Linoleic acid has an effective role in lowering the level of lipids in the blood as well as helping in the reduction of arthritis [41].

### 2.5. Carbohydrate

It has been noted that edible mushrooms contain large amounts of carbohydrates as they contain a large proportion of glucose and mannitol and in return contain small amounts of sucrose and fructose [42]. Mushrooms are rich in dietary fibers because of nonstarch polysaccharides. Stems of mushrooms are a great source of insoluble dietary fibers. Mushrooms contain about 4% to 9% of soluble fibers as well as 22% to 30% of insoluble fibers. Previous studies have revealed that 100 grams of *P. ostreatus* mushroom contain 4.1 grams of dietary fibers [43].

### 2.6. Vitamins

Edible mushroom fruit body contains vitamins especially B1, B2, C, E, and D2. The B vitamins such as thiamine, riboflavin, pyridoxine, pantothenic acid, nicotinic acid, nicotinamide, folic acid, and cobalamin have been the most frequently observed. The species *P. ostreatus* contains folacin, vitamin B1, and vitamin B3 in the greater amount [44].

## 3. Health Benefit of Edible Mushroom

Edible mushrooms have been revered for their immense health benefits. Specific biochemical compounds in mushrooms are responsible for improving human health in many ways. These bioactive compounds include polysaccharides, triterpenoids, low molecular weight proteins, glycoproteins, and immunomodulating compounds. Hence, mushrooms have been shown to promote immune function; boost health; lower the risk of cancer; inhibit tumor growth; help balancing blood sugar; ward off viruses, bacteria, and fungi; reduce inflammation; and support the body's detoxification mechanisms. Increasing recognition of mushrooms in complementing conventional medicines is also well known for fighting many diseases. Medicinal values of some important mushroom are shown in Table 2.

### 3.1. Antimicrobial Activity

Edible mushrooms have an antibacterial effect given the fact that they contain significant amounts of phenols and alkaloids. Several studies have shown that mushrooms are effective against many different strains of bacterial pathogens that cause various diseases to humans. The edible mushrooms that have antimicrobial activity include *L. delicious, S. imbricatus,* and *T. portentosum*. On the other hand, the exudate material collected from the mycelium body of several mushroom species is biologically active against plasmodium falciparum, the major cause of malaria that is transmitted by the female mosquito. Up to the present, malaria remains the number one cause of death among children aged below five in the majority of countries in sub-Saharan Africa [60].

The extracts of Ganoderma pfeifferi inhibit the growth of microorganisms responsible for skin problems [61]. Oxalic acid has been found to be the compound responsible for the antimicrobial effect of Lentinula edodes (Berk.) against *S. aureus* and other bacteria [62]. Ethanoic mycelial extracts from *L. edodes* possess antiprotozoal activity against paramecium caudatum [63].

Edible mushrooms have been noted to combat various microorganisms and kill them. *L. sulphuerus* is, for example, another type of edible mushroom that contains flavonoid, phenol, lycopene, and ascorbic acid that possess potential antimicrobial activity [64]. Mushrooms have secondary compounds such as triterpene which plays an effective role as an antiviral agent, especially for HIV [65]. It has been shown that *P. ostreatus* acts as a broad-spectrum antibacterial substance [66]. *Staphylococcus*, *Bacillus* subtilis, *Streptococcus mutants, P. intermedia*, and *E. coli* have been reported to be killed by chloroform and ethyl acetate extract of mushroom [67].

### 3.2. Anticancer Activity

Studies have proven that edible mushrooms have anticancer activity, especially in cases of liver, uterine, breast, and pancreatic cancer. This is because it contains secondary metabolites such as terpenoids, which are known for their anticancer activity. Also, the presence of P-glucan works to inhibit breast cancer cells. In one of the studies, it was found that mushrooms have proven cancer activity in cancerous liver cells by stopping the proliferation according to while it worked to inhibit tumor growth [68]. In addition, mushrooms contain large amounts of glucans ranging from 0.21 to 0.53 grams per 100 grams of dry weight, as they are the main component of the fungal cell, and *β*-glucans are known to have anticancer, cholesterol-lowering, and immune system-stimulating effects that fight against cancer cells [69].

### 3.3. Antimutagenic Activity

Edible mushrooms contain biological compounds with wide antimutagenic and immune-modulatory activities. Amongst their bioactive compounds, beta-glucan is considered as glucose residual backbone linked by a beta-1-3-glycosidic bond with attached beta-1-6 branch point which plays antitumor, immunomodulatory, and immune-stimulant activity roles. Due to its immunomodulatory activity, it activates and stimulates the natural killer cells: neutrophils, macrophages, T-cells, and B-cells that work against cancerous cells and lyse them [70]. Polysaccharide extract of mushrooms plays radical scavenging activity in addition to antitumor activity. About eight species of mushroom have been investigated for whose polysaccharide extract plays an antitumor activity by scavenging free radicals such as superoxide and hydroxyl radicals [68].

### 3.4. Antioxidant Activity

Edible mushrooms are rich in antioxidants due to their containment of polysaccharides [13]. It has been revealed from studies that mushrooms containing phenolic extract have great potential as antioxidant. The antioxidant activity is majorly found in the fruiting body of mushrooms because they have phenolic acid and phenolic compounds in methanol extract of them. Among mushrooms, *Fomitotpsispinicola* and *Gloeophyllumsepiarium* have strong antioxidant activity. In some types of mushrooms, beta-carotene and linoleic acid are also found which induce inhibition of auto-oxidation [71]. Mushrooms also contain ergosterol, which is a precursor for the manufacture of vitamin D and is known for its effective role in antioxidant properties [72]. A study of methanolic extracts from black, red, and snow ear mushrooms found that mushrooms possess an inhibitory effect on lipid peroxidation, 1,1-diphenyl-2-picrylhydrazyl (DPPH) radical scavenging and hydroxyl radical scavenging, and a strong reducing power and ability to chelate ferrous ions [73]. Similar studies on other mushrooms, including *D. indusiata, G. frondosa, H. erinaceus, T. giganteum, F. velutipes, L. edodes, P. cystidiosus,* and *P. ostreatus*, Agrocybe cylindracea also reported antioxidant properties of these mushrooms [74].

## 4. Conclusion

Edible mushrooms are valuable resources for food and medicine. The most common nutrients in edible mushrooms are protein, carbohydrates, amino acids, fatty acids, and vitamins. Edible mushrooms have antimicrobial properties that prevent and reduce different diseases by releasing antioxidants directly associated with various diseases. The medicinal properties of mushrooms include anti-inflammatory, antioxidant, immunomodulatory, anticarcinogenic, antiviral, antibacterial, antifungal, hepato-protective, antidiabetic, antiangiogenic, and hypoglycemic. This would greatly facilitate the application of edible mushrooms as functional food ingredients or products that could provide various health benefits to humans in the future.

Knowledge on dose requirement, route and timing of administration, mechanism of action, and site of activity is lacking. If these challenges are met out in the coming days, mushroom industries will play a lead role in nutraceutical and pharmaceutical industries. The increasing awareness about high nutritional value accompanied by medicinal properties means that mushrooms are going to be important food item in coming days and at places may emerge as an alternate to nonvegetarian foods. Growing mushroom is economically and ecologically beneficial. Consuming mushroom is beneficial in every respect.

Future studies into the mechanisms of action of mushroom extracts will help us to further delineate the interesting roles and properties of various mushroom phytochemicals in the prevention and treatment of some degenerative diseases. In view of the current situation, the research of bioactive components in edible wild and cultivated mushrooms is yet deficient. There are numerous potential characteristics and old and novel properties, provided by mushrooms with nutraceutical and health benefits, which deserve further investigations.

## Figures and Tables

**Table 1:** Nutritional value of some commercial edible mushrooms (on dry wt. basis) [37].

Nutritional parameters	Mushrooms			
	*Agaricus bisporus*	*Pleurotus spp.*	*Volvariella volvacea*	*Lentinula edodes*
Proteins %	29.14	19.59	38.10	18.85
Carbohydrates %	51.05	64.34	42.30	63.60
Fat %	1.56	1.05	0.97	1.22
Vitamins D (IU/g)	984	487	462.04	205
Sodium (mg/kg)	500.8	208.87	345.34	82.49
Iron (mg/kg)	85.86	183.07	72.51	37.55
K : Na	84 : 1	129 : 1	120 : 1	255 : 1

**Table 2:** Medicinal values of some important mushrooms.

Mushroom	Compounds	Medicinal properties	Authors
*Lyophyllum shimeji*	A novel fibrinolytic enzyme: *α*-chymotrypsin	Blood anticoagulant	[45]
*Lentinula edodes*	Polysaccharides	Antioxidant	[46]
*Phallus indusiatus*	A *β*-D-glucan called T-5-N	Anti-inflammatory properties	[47]
		Antioxidant capability	[48]
*Pleurotus ostreatus*	Lovastatin: inhibitor of 3-hydroxy-3-methylglutaryl coenzyme A reductase	Reduction of cholesterol	[46]
	Oyster mushroom concentrate	Anti-inflammatory activity	[49]
*Pleurotus eryngii*	Acidic glycosphingolipids	Antitumour activity; immune system enhancer; antibacterial activity	[50]
*Hericium erinaceus*	Glycoprotein HEG-5	Hemagglutinating activity	[51]
	Polysaccharides (HEPs)	Antibacterial activity against *Helicobacter pylori*	[52]
	Glycoprotein HEG-5	Anticancer potential against human gastrointestinal cancers	[53]
*Crucibulum leave*	A new salfredin-type metabolites (DSM 1653 and DSM 8519	Inhibition of the enzyme aldose reductase	[54]
*Lyophyllum shimeji*	A novel fibrinolytic enzyme: *α*- chymotrypsin	Blood anticoagulant	[45]
*Agaricus bisporus*	Gallic acid, protocatechuic acid, catechin, caffeic acid, ferulic acid and myricetin	Antioxidant activity	[55]
		Immune system enhancer	[56]
		Anticancer	[57]
*Hydnellum peckii*	(2,5-dihydroxy-3,6-bis (4-hydroxyphenyl)-1,4-benzoquinone)	Anticoagulant	[58]
		Antibacterial activity atromentin and leucomelone	[59]

## Data Availability

The data used to support the findings of this review are included within the article.

## References

[1] Chang S.-T., Miles P. G. (2004). *Mushrooms: Cultivation, Nutritional Value, Medicinal Effect, and Environmental Impact*.

[2] Ogidi C. O., Oyetayo V. O., Akinyele B. J. (2020). Wild medicinal mushrooms: potential applications in phytomedicine and functional foods. *An Introduction to Mushroom*.

[3] Chang S.-T. (2006). The world mushroom industry: trends and technological development. *International Journal of Medicinal Mushrooms*.

[4] Rahi D. K., Malik D. (2016). Diversity of mushrooms and their metabolites of nutraceutical and therapeutic significance. *The Journal of Mycology*.

[5] Kumla J., Suwannarach N., Sujarit K. (2020). Cultivation of mushrooms and their lignocellulolytic enzyme production through the utilization of agro-industrial waste. *Molecules*.

[6] Thu Z. M., Myo K. K., Aung H. T., Clericuzio M., Armijos C., Vidari G. (2020). Bioactive phytochemical constituents of wild edible mushrooms from Southeast Asia. *Molecules*.

[7] Falconer J., Koppell C. R. (1990). The major significance of minor’ forest products. *The Local Use and Value of Forests in the West African Humid Forest Zone*.

[8] Degreef J., Malaisse F., Rammeloo J., Baudart E. (1997). A nutritional and ecological approach. *Biotechnology, Agronomy, Society and Environment*.

[9] Vinceti B., Termote C., Ickowitz A., Powell B., Kehlenbeck K., Hunter D. (2013). The contribution of forests and trees to sustainable diets. *Sustainability*.

[10] Hossain M. S., Alam N., Amin S. M., Basunia M. A. (2007). Essential fatty acid contents of Pleurotus ostreatus, Ganoderma lucidum and Agaricus bisporus. *Bangladesh J Mushroom*.

[11] Alam N., Khan M. A., Hossain M. S., Amin S. M. R. (2007). Nutritional analysis of dietary mushroom Pleurotus Florida eger and Pleurotus sajor-caju (Fr.) singer. *Bangladesh Journal of Mushroom*.

[12] Sánchez C. (2004). Modern aspects of mushroom culture technology. *Applied Microbiology and Biotechnology*.

[13] Seo D. J., Choi C. (2021). Antiviral bioactive compounds of mushrooms and their antiviral mechanisms: a review. *Viruses*.

[14] Côté J., Caillet S., Doyon G., Sylvain J. F., Lacroix M. (2010). Bioactive compounds in cranberries and their biological properties. *Critical Reviews in Food Science and Nutrition*.

[15] D’Archivio M., Filesi C., Vari R., Scazzocchio B., Masella R. (2010). Bioavailability of the polyphenols: status and controversies. *International Journal of Molecular Sciences*.

[16] Venturella G., Ferraro V., Cirlincione F., Gargano M. L. (2021). Medicinal mushrooms: bioactive compounds, use, and clinical trials. *International Journal of Molecular Sciences*.

[17] Javed S., Li W. M., Zeb M. (2019). Anti-inflammatory activity of the wild mushroom, echinodontium tinctorium, in RAW264.7 macrophage cells and mouse microcirculation. *Molecules*.

[18] Kany S., Vollrath J. T., Relja B. (2019). Cytokines in inflammatory disease. *International Journal of Molecular Sciences*.

[19] Younis A. M., Yosri M., Stewart J. K. (2019). In vitro evaluation of pleiotropic properties of wild mushroom Laetiporus sulphureus. *Annals of Agricultural Science*.

[20] Palacios I., Lozano M., Moro C. (2011). Antioxidant properties of phenolic compounds occurring in edible mushrooms. *Food Chemistry*.

[21] Manzi P., Aguzzi A., Pizzoferrato L. (2001). Nutritional value of mushrooms widely consumed in Italy. *Food Chemistry*.

[22] Sánchez C. (2010). Cultivation of Pleurotus ostreatus and other edible mushrooms. *Applied Microbiology and Biotechnology*.

[23] Dundar A., Acay H., Yildiz A. (2008). Yield performances and nutritional contents of three oyster mushroom species cultivated on wheat stalk. *African Journal of Biotechnology*.

[24] Kalač P. (2009). Chemical composition and nutritional value of European species of wild growing mushrooms: a review. *Food Chemistry*.

[25] Heleno S. A., Barros L., Sousa M. J., Martins A., Ferreira I. C. F. R. (2010). Tocopherols composition of Portuguese wild mushrooms with antioxidant capacity. *Food Chemistry*.

[26] Mattila P., Könkö K., Eurola M. (2001). Contents of vitamins, mineral elements, and some phenolic compounds in cultivated mushrooms. *Journal of Agricultural and Food Chemistry*.

[27] Barros L., Cruz T., Baptista P., Estevinho L. M., Ferreira I. C. F. R. (2008). Wild and commercial mushrooms as source of nutrients and nutraceuticals. *Food and Chemical Toxicology*.

[28] Pehrsson P. R., Haytowitz D. B., Holden J. M. (2003). The USDA’s national food and nutrient analysis program: update 2002. *Journal of Food Composition and Analysis*.

[29] Ferreira I., Barros L., Abreu R. (2009). Antioxidants in wild mushrooms. *Current Medicinal Chemistry*.

[30] Pereira E., Barros L., Martins A., Ferreira I. C. F. R. (2012). Towards chemical and nutritional inventory of Portuguese wild edible mushrooms in different habitats. *Food Chemistry*.

[31] Vaz J. A., Heleno S. A., Martins A., Almeida G. M., Vasconcelos M. H., Ferreira I. C. F. R. (2010). Wild mushrooms Clitocybe alexandri and Lepista inversa: in vitro antioxidant activity and growth inhibition of human tumour cell lines. *Food and Chemical Toxicology*.

[32] Rathee S., Rathee D., Rathee D., Kumar V., Rathee P. (2012). Mushrooms as therapeutic agents. *Revista Brasileira de Farmacognosia*.

[33] Wiseman H., O’Reilly J., Lim P., Garnett A. P., Huang W. C. (1998). *Functional Foods, the Consumer, the Products and the Evidence*.

[34] Bellettini M. B., Fiorda F. A., Maieves H. A. (2019). Factors affecting mushroom Pleurotus spp. *Saudi Journal of Biological Sciences*.

[35] Gupta S., Summuna B., Gupta M., Annepu S. K. (2018). Edible mushrooms: cultivation, bioactive molecules, and health benefits. *Bioactive Molecules in Food*.

[36] Sławińska A., Jabłońska-Ryś E., Stachniuk A. (2021). High-performance liquid chromatography determination of free sugars and mannitol in mushrooms using corona charged aerosol detection. *Food Analytical Methods*.

[37] Ahlawat O., Manikandan K., Singh M. (2016). Proximate composition of different mushroom varieties and effect of UV light exposure on vitamin D content in Agaricus bisporus and Volvariella volvacea. *Mushroom Res*.

[38] Longvah T., Deosthale Y. G. (1998). Compositional and nutritional studies on edible wild mushroom from northeast India. *Food Chemistry*.

[39] Yilmaz N., Solmaz M., Türkekul İ., Elmastaş M. (2006). Fatty acid composition in some wild edible mushrooms growing in the middle Black Sea region of Turkey. *Food Chemistry*.

[40] Pedneault K., Angers P., Gosselin A., Tweddell R. J. (2006). Fatty acid composition of lipids from mushrooms belonging to the family Boletaceae. *Mycological Research*.

[41] Podkowa A., Kryczyk-Poprawa A., Opoka W., Muszyńska B. (2020). Culinary–medicinal mushrooms: a review of organic compounds and bioelements with antioxidant activity. *European Food Research and Technology*.

[42] Uddin P. K. M., Pervin R., Jahan J., Talukder R. I., Ahmed S., Rahman M. (2020). Mushroom polysaccharides: chemistry and anticancer potentials. *An Introduction to Mushroom*.

[43] Ma Q. L., Zhu C., Morselli M. (2020). The novel omega-6 fatty acid docosapentaenoic acid positively modulates brain innate immune response for resolving neuroinflammation at early and late stages of humanized APOE-based Alzheimer’s disease models. *Frontiers in Immunology*.

[44] Tagkouli D., Kaliora A., Bekiaris G. (2020). Free amino acids in three Pleurotus species cultivated on agricultural and agro-industrial by-products. *Molecules*.

[45] Moon S.-M., Kim J.-S., Kim H.-J. (2014). Purification and characterization of a novel fibrinolytic *α* chymotrypsin like serine metalloprotease from the edible mushroom, Lyophyllum shimeji. *Journal of Bioscience and Bioengineering*.

[46] Chen H., Ju Y., Li J., Yu M. (2012). Antioxidant activities of polysaccharides from Lentinus edodes and their significance for disease prevention. *International Journal of Biological Macromolecules*.

[47] Hara C., Kiho T., Tanaka Y., Ukai S. (1982). Anti-inflammatory activity and conformational behavior of a branched (1⟶3)-*β*-d-glucan from an alkaline extract of dictyophora indusiata fisch. *Carbohydrate Research*.

[48] Ker Y.-B., Chen K.-C., Peng C.-C., Hsieh C.-L., Peng R. Y. (2011). Structural characteristics and antioxidative capability of the soluble polysaccharides present in Dictyophora indusiata (Vent. Ex Pers.) Fish Phallaceae. *Evidence-Based Complementary and Alternative Medicine*.

[49] Jedinak A., Dudhgaonkar S., Wu Q. L., Simon J., Sliva D. (2011). Anti-inflammatory activity of edible oyster mushroom is mediated through the inhibition of NF-*κ*B and AP-1 signaling. *Nutrition Journal*.

[50] Nozaki H., Itonori S., Sugita M. (2008). Mushroom acidic glycosphingolipid induction of cytokine secretion from murine T cells and proliferation of NK1.1 *α*/*β* TCR-double positive cells in vitro. *Biochemical and Biophysical Research Communications*.

[51] Cui F.-J., Li Y.-H., Zan X.-Y. (2014). Purification and partial characterization of a novel hemagglutinating glycoprotein from the cultured mycelia of Hericium erinaceus. *Process Biochemistry*.

[52] Zhu Y., Chen Y., Li Q. (2014). Preparation, characterization, and anti-Helicobacter pylori activity of Bi3+-Hericium erinaceus polysaccharide complex. *Carbohydrate Polymers*.

[53] Li G., Yu K., Li F. (2014). Anticancer potential of Hericium erinaceus extracts against human gastrointestinal cancers. *Journal of Ethnopharmacology*.

[54] Neumann T., Schlegel B., Hoffmann P., Heinze S., Graefe U. (1999). Isolation and structure elucidation of new salfredin‐type metabolites from Crucibulum laeve DSM 1653 and DSM 8519. *Journal of Basic Microbiology: An International Journal on Biochemistry, Physiology, Genetics, Morphology, and Ecology of Microorganisms*.

[55] Liu J., Jia L., Kan J., Jin C.-h. (2013). In vitro and in vivo antioxidant activity of ethanolic extract of white button mushroom (Agaricus bisporus). *Food and Chemical Toxicology*.

[56] Ren Z., Guo Z., Meydani S. N., Wu D. (2008). White button mushroom enhances maturation of bone marrow-derived dendritic cells and their antigen presenting function in mice. *Journal of Nutrition*.

[57] Zhang M., Huang J., Xie X., Holman C. D. A. J. (2009). Dietary intakes of mushrooms and green tea combine to reduce the risk of breast cancer in Chinese women. *International Journal of Cancer*.

[58] Khanna J. M., Malone M. H., Euler K. L., Brady L. R. (1965). Atromentin anticoagulant from hydnellum diabolus. *Journal of Pharmaceutical Sciences*.

[59] Zheng C.-J., Sohn M.-J., Kim W.-G. (2006). Atromentin and leucomelone, the first inhibitors specific to enoyl-ACP reductase (FabK) of Streptococcus pneumoniae. *Journal of Antibiotics*.

[60] Rahimah S. B., Djunaedi D. D., Soeroto A. Y., Bisri T. (2019). The the phytochemical screening, total phenolic contents and antioxidant activities in vitro of white oyster mushroom (Pleurotus ostreatus) preparations. *Open access Macedonian journal of medical sciences*.

[61] Mothana R. A. A., Jansen R., Jülich W.-D., Lindequist U. (2000). Ganomycins A and B, new antimicrobial farnesyl hydroquinones from the basidiomycete Ganoderma pfeifferi. *Journal of Natural Products*.

[62] Bender S., Dumitrache-Anghel C. N., Backhaus J. (2003). A case for caution in assessing the antibiotic activity of extracts of culinary-medicinal Shiitake mushroom [Lentinus edodes (Berk.) Singer](Agaricomycetideae). *International Journal of Medicinal Mushrooms*.

[63] Badalyan S. M. (2004). Antiprotozoal activity and mitogenic effect of mycelium of culinary-medicinal Shiitake mushroom Lentinus edodes (Berk.) Singer (Agaricomycetideae). *International Journal of Medicinal Mushrooms*.

[64] Mickymaray S. (2019). Efficacy and mechanism of traditional medicinal plants and bioactive compounds against clinically important pathogens. *Antibiotics*.

[65] Złotko K., Wiater A., Waśko A. (2019). A report on fungal (1⟶3)-*α*-d-glucans: properties, functions and application. *Molecules (Basel, Switzerland)*.

[66] Balaky S. T. J., Mawlood A. H., Shabila N. P. (2019). Survival analysis of patients with tuberculosis in Erbil, Iraqi Kurdistan region. *BMC Infectious Diseases*.

[67] Parham S., Kharazi A. Z., Bakhsheshi-Rad H. R. (2020). Antioxidant, antimicrobial and antiviral properties of herbal materials. *Antioxidants*.

[68] Del Cornò M., Gessani S., Conti L. (2020). Shaping the innate immune response by dietary glucans: any role in the control of cancer?. *Cancers*.

[69] Derbyshire E. J. (2020). Is there scope for a novel mycelium category of proteins alongside animals and plants?. *Foods*.

[70] Osman A., Toliba A. O. (2019). Hepatoprotective effects of crude phenolic-rich extract from oyster mushroom (Pleurotus ostreatus). *Egyptian Journal of Food Science*.

[71] Karácsonyi Š., Kuniak Ľ. (1994). Polysaccharides of Pleurotus ostreatus: isolation and structure of pleuran, an alkali-insoluble *β*-D-glucan. *Carbohydrate Polymers*.

[72] Zeb M., Lee C. H. (2021). Medicinal properties and bioactive compounds from wild mushrooms native to North America. *Molecules*.

[73] Kasuga A., Aoyagi Y., Sugahara T. (1993). Antioxidative activities of several mushroom extracts. *Nippon Shokuhin Kogyo Gakkaishi*.

[74] Mau J.-L., Lin H.-C., Song S.-F. (2002). Antioxidant properties of several specialty mushrooms. *Food Research International*.

